# Radiofrequency ablation for recurrent hepatocellular carcinoma: 10-year outcomes of local tumor progression vs intrahepatic distant recurrence

**DOI:** 10.1186/s13244-025-02080-9

**Published:** 2025-09-16

**Authors:** Huan-ling Guo, Jia-qian Yao, Xin Zheng, Tong-yi Huang, Xiao-er Zhang, Rui Zhang, Wen-xin Wu, Xiao-yan Xie, Ming Xu

**Affiliations:** Department of Medical Ultrasonics, Institute of Diagnostic and Interventional Ultrasound, The First Affiliated Hospital of Sun Yat-Sen University, Guangzhou, China

**Keywords:** Carcinoma (hepatocellular), Radiofrequency ablation, Neoplasm recurrence (local), Neoplasm recurrence, Distant, Survival

## Abstract

**Objectives:**

To compare the long-term outcome of radiofrequency ablation (RFA) for local tumor progression (LTP) vs intrahepatic distant recurrence (IDR) in recurrent hepatocellular carcinoma (rHCC), including cases with repeated LTP.

**Materials and methods:**

From 2010 to 2022, 1326 rHCC patients treated with curative-intent RFA were identified. Propensity score matching (PSM) was used to balance the bias between the LTP group and the IDR group. Overall survival (OS) and progression-free survival (PFS), were compared between groups using log-rank tests and Cox proportional hazards models.

**Results:**

A total of 584 patients were finally enrolled (125 LTPs, 459 IDRs), with a median follow-up of 5.8 years. After PSM, 218 patients (109 patients in each group) were selected. The median OS was comparable between LTP and IDR (70.3 months vs 93.1 months, *p* = 0.974). However, PFS was significantly worse in the LTP group (13.8 months vs 20.9 months, *p* = 0.028). LTP incidence was higher in the LTP group (42.2% vs 12.8%, *p* < 0.001). Multiple recurrences, early recurrence (≤ 1 year), and ≥ 3 LTP episodes were independent risk factors for OS. The median OS decreased with increasing LTP episodes (0: 99.3 months; 1: 86.9 months; 2: 88.9 months; ≥ 3: 44.9 months, *p* = 0.031).

**Conclusions:**

RFA demonstrated effective control of LTP in rHCC, with comparable OS but worse PFS compared with IDR, primarily due to the higher risk of LTP. RFA may not be the first choice for those with ≥ 3 LTP episodes.

**Critical relevance statement:**

LTP of hepatocellular carcinoma shows higher recurrence than IDR after RFA, requiring close follow-up. Three or more repeat LTPs significantly worsen prognosis, suggesting the need for alternative treatment strategies.

**Key Points:**

- Long-term outcomes of RFA for LTP vs IDR of hepatocellular carcinoma remain unclear.
- LTP has worse PFS; ≥ 3 repeat LTP significantly worsens OS.
- LTP tends to recur after RFA, requiring close follow-up; ≥ 3 repeat LTPs need alternative local treatment.

**Graphical Abstract:**

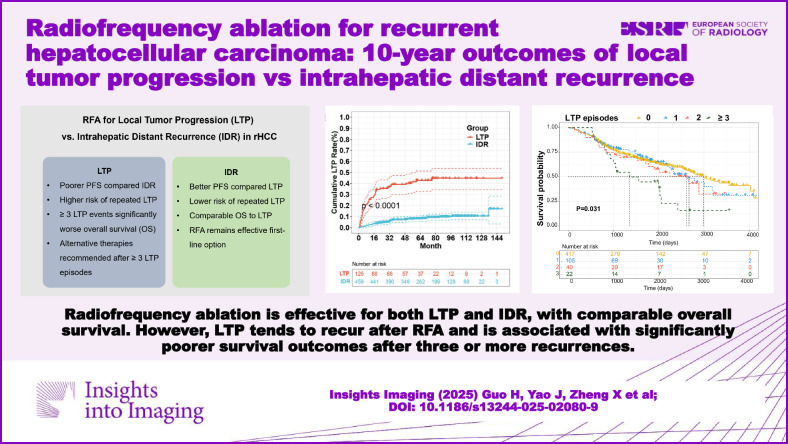

## Introduction

Hepatocellular carcinoma (HCC) is the fourth leading cause of cancer death worldwide [1]. Thermal ablation achieves tumor inactivation through energy transfer and localized tissue heating, offering comparable efficacy with advantages over surgical resection, including reduced invasiveness, quicker recovery, and broader applicability [2]. Consequently, numerous guidelines endorse it as a first-line option for early-stage HCC [3]. Nevertheless, thermal ablation is associated with higher rates of local tumor progression (LTP), reported in 2–60% of cases [4, 5], which some studies suggest may adversely impact patient survival [6].

Radiofrequency ablation (RFA) has been demonstrated as a comparable alternative to repeat resection for rHCC, owing to its precise ablation margin control and favorable safety profile [7–9]. LTP is frequently observed in technically demanding scenarios, including subcapsular, perivascular, or perivisceral locations [4], where RFA is often preferred due to its adaptability and precision in electrode placement [10]. Nevertheless, the biological characteristics of LTP differ from those of intrahepatic distant recurrence (IDR), potentially leading to distinct prognoses. LTP has been shown to exhibit more aggressive biological features, including enhanced proliferation, invasiveness, epithelial-mesenchymal transition, and thermal resistance [11–13], potentially rendering it less responsive to RFA compared to IDR. These suggest that the indications, treatment protocols, and follow-up strategies for RFA in LTP may need to differ from those for IDR to achieve better local control. To date, only four studies have evaluated the efficacy of RFA for LTP, and consistently reported that repeated RFA for treating first-time LTP achieves comparable disease-free survival and overall survival (OS) outcomes to surgery [14, 15] or Transarterial Chemoembolization (TACE) [16, 17], with moderate local control rates of 78.3–90.3% [15, 17]. However, comparative data evaluating the outcomes of RFA between LTP and IDR are lacking. Additionally, the long-term outcomes of repeated RFA for the same-site recurrent LTP, even when technically feasible, are not well established. Moreover, the optimal timing for switching to alternative therapies has also not been previously assessed.

Therefore, this study aimed to compare the long-term survival outcomes of RFA between LTP and IDR, as well as to comprehensively identify prognostic factors of RFA for rHCC, focusing on the long-term implications for recurrent LTP.

## Materials and methods

### Ethics statement

All investigations were carried out in compliance with the ethical principles outlined in both the Helsinki and Istanbul Declarations. This study received ethical approval from the First Affiliated Hospital of Sun Yat-sen University (no. [2023]770), with waived informed consent.

### Patients

**Fig. 1:**
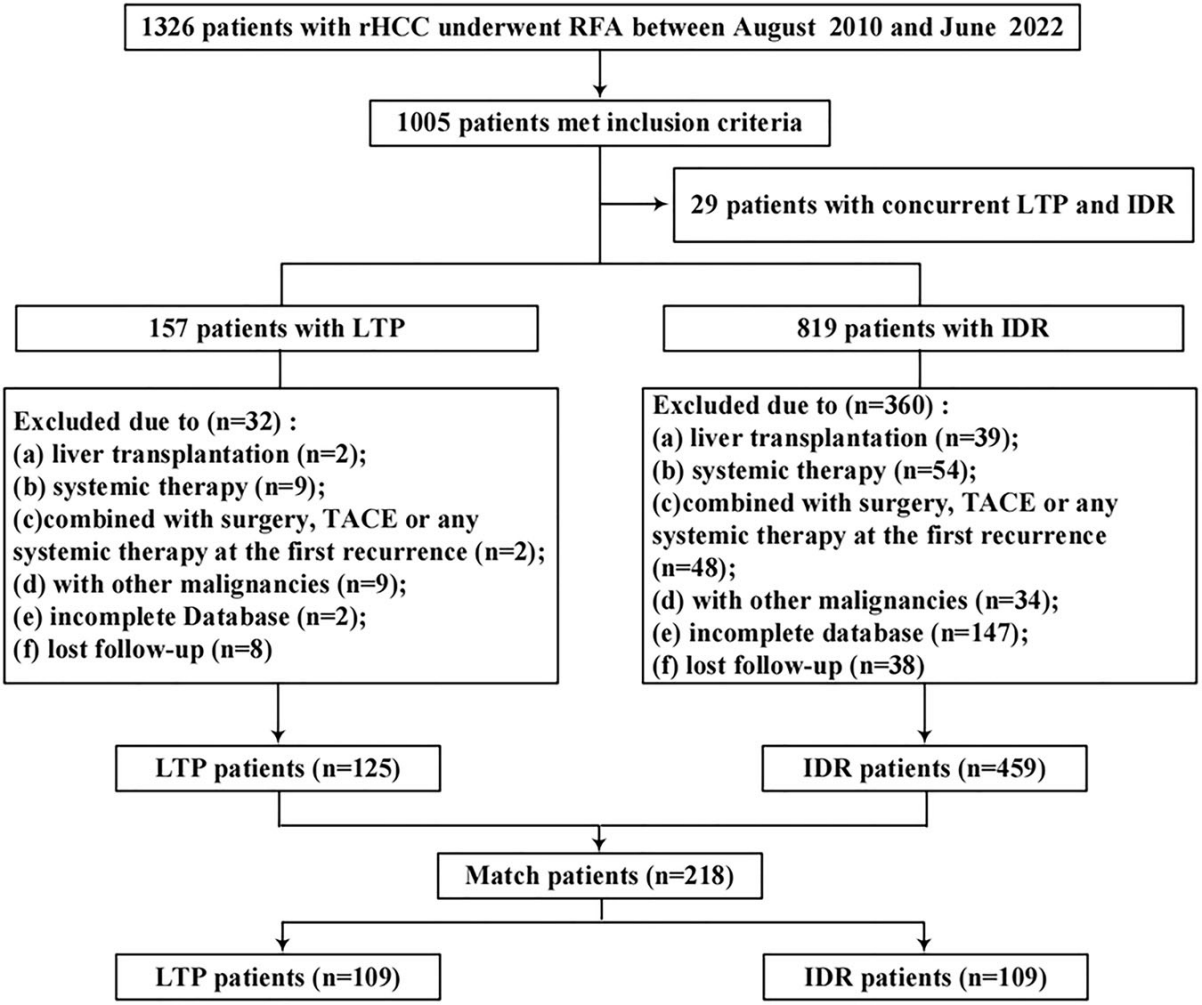
Flow diagram of patient selection. rHCC, recurrent hepatocellular carcinoma; RFA, radiofrequency ablation; LTP, local tumor progression; IDR, intrahepatic distant recurrence; TACE, transarterial chemoembolization

This study was a retrospective analysis of data from a high-volume center. Consecutive rHCC patients who underwent curative-intent percutaneous US-guided RFA from August 30th, 2010, to June 30th, 2022, were recruited (Fig. 1).

The inclusion criteria for rHCC patients were as follows: (a) age ≥ 18 years; (b) single HCC ≤ 5 cm or up to 3 tumors ≤ 3 cm without vascular invasion or extrahepatic metastasis (for curative treatment), according to the Milan criteria; (c) Child–Pugh class A or B; (d) Eastern Cooperative Oncology Group (ECOG) performance status (PS) 0 or 1; and (e) complete RFA. The corresponding exclusion criteria were as follows: (a) concurrent LTP and IDR; (b) combination with other malignancies; (c) history of liver transplantation or any systemic therapy before thermal ablation at recurrence; (d) combined surgery, transarterial chemoembolization (TACE), or any systemic therapy at recurrence; (e) incomplete clinicopathological data; and (f) lost to follow-up.

### Diagnosis and treatment choice

RHCC was diagnosed based on histological findings, or to the European Association for the Study of the Liver, European Organization for Research and Treatment of Cancer (EASL-EORTC) guidelines [18]. LTP was defined as lesions occurring within 1 cm of the ablation zone more than 1 month after complete ablation (confirmed by contrast-enhanced imaging, including contrast-enhanced ultrasound (US) combined with either contrast-enhanced CT or MRI), while intrahepatic rHCC beyond 1 cm was classified as IDR [19–21].

The decision to perform RFA was made following consensus from an interdisciplinary conference comprising experts in liver surgery, US intervention, radiology, and oncology, who considered liver function, tumor status, health status, and technical feasibility. The patients made the final decision after understanding the possible efficacy, cost, and risk of RFA complications.

### RFA procedure

All RFA procedures were performed by experienced radiologists (X.-y.X., M.X., and X.-e.Z.), under real-time US guidance. Most patients received subcutaneous anesthesia with intravenous analgesia, while patients > 80 years, or those with cardiac or pulmonary issues, received intravenous anesthesia. Vital signs were monitored continuously. The Cool-tip^TM^ RFA system (Valleylab, Boulder, CO, USA) was utilized with 17-gauge electrodes, and the number of ablation points and duration were tailored to the tumor’s characteristics to ensure a ≥ 0.5 cm margin. Combination with percutaneous ethanol injection (PEI) was adopted as necessary in high-risk lesions, defined as tumors within 3 mm of the capsule (subcapsular), a vital structure, or a blood vessel 3 mm in diameter or larger [22–25]. The electrode path was cauterized following RFA.

The ablated zone was assessed 30 min, one day, and one month after RFA using contrast-enhanced ultrasound (CEUS). Supplementary ablation with RFA or PEI was performed until complete ablation was achieved, if ablation was incomplete.

### Follow-up

The initial follow-up was conducted one month after the initial thermal ablation treatment, with subsequent assessments every three months until death or dropout. During each follow-up visit, serum AFP levels, liver function tests, CEUS, and contrast-enhanced CT/MRI scans were conducted.

Follow-up data were collected through multiple channels, encompassing telephone interviews, in-person visits, clinical documentation, and mortality records. Progression-free survival (PFS) was defined as the interval from curative RFA for rHCC to intrahepatic recurrence, extrahepatic spread, death, or the follow-up endpoint (December 7th, 2023). OS was defined as the time from the initial RFA to death or the follow-up endpoint.

### Statistical analysis

Propensity score matching (PSM, 1:1 nearest-neighbor, caliper = 0.02) was performed using significant baseline variables identified via Cox regression. Sample size was calculated based on the primary outcome of the median PFS [26] in our data using PASS version 15.0, with 91 patients required to detect a significant difference (α = 0.05, power = 90%) between LTP (14 months) and IDR (23 months); Both the entire cohort (125 LTPs and 459 IDRs; estimated power = 99.67%) and the matched cohort (109 patients per group; estimated power = 90.41%) exceeded the required sample size threshold. Kaplan–Meier curves were generated for both unadjusted and PSM-adjusted cohorts, with PFS and OS compared using the log-rank test. Cox proportional hazards models were used for univariate and multivariate analyses (*p* < 0.1 for inclusion), and the proportional hazards assumption was verified via Schoenfeld residuals (*p* > 0.05). Continuous variables were reported as mean ± SD and compared using *t*-tests or ANOVA; categorical variables were analyzed using χ² or Fisher’s exact tests. Statistical significance was defined as *p* < 0.05.

## Results

### Patient characteristics

The initial data review identified 1326 patients, of whom 584 eligible patients were finally enrolled and divided into two groups based on the location of rHCC: the LTP group (*n* = 125) and the IDR group (*n* = 459) (Fig. 1). Analysis of baseline characteristics, detailed in Table 1, revealed no significant differences in sex, age, HBV and HCV infections, antiviral therapy, cirrhosis, portal hypertension, Child–Pugh score, or serum levels of alanine aminotransferase, aspartate aminotransferase, or alpha fetoprotein between the groups. There was no significant difference in tumor size between the groups; however, the IDR group exhibited a significantly higher proportion of multiple tumors (20.0% vs 11.2%; *p* = 0.032). The number of subcapsular lesions (within 3 mm of the capsule) was significantly higher in the LTP group (43.2% vs 28.8%; *p* = 0.003). A total of 372 IDRs (81.0%) and 46 LTPs (36.8%) recurred in primary HCC, whereas 87 IDRs (19.0%) and 79 LTPs (63.2%) experienced rHCC recurrence (*p* < 0.001). Regarding prior treatment, 85 (18.5%), 329 (71.7%), and 45 (9.8%) patients with IDR and 57 (45.6%), 57 (45.6%), and 11 (8.8%) patients with LTP previously underwent thermal ablation, partial hepatectomy, and TACE for primary HCC, respectively (*p* < 0.001).

**Table 1:** Baseline characteristics of patients with rHCC before RFA

Variable	Before PSM adjustment			After PSM adjustment		
	IDR	LTP	*p* value	IDR	LTP	*p* value
No. of patients	459	125	…	109	109	…
Sex (male)	400 (87.1)	110 (88.0)	0.918	91 (83.5)	95 (87.2)	0.566
Age (≥ 60 years)	181 (39.4)	60 (48.0)	0.105	45 (41.3)	55 (50.5)	0.221
HBV (positive)	435 (95.2)	120 (96.0)	0.886	104 (96.3)	105 (96.3)	1
HCV (positive)	8 (1.7)	3 (2.4)	0.914	0 (0.0)	3 (2.8)	0.245
Anti-hepatitis therapy (yes)	227 (49.5)	65 (52.0)	0.687	64 (58.7)	59 (54.1)	0.585
Cirrhosis (yes)	255 (55.6)	65 (52.0)	0.544	64 (58.7)	64 (58.7)	1
Portal hypertension (yes)	147 (32.0)	32 (25.6)	0.203	32 (29.4)	29 (26.6)	0.763
Child–Pugh grade			0.742			0.879
A	329 (71.6)	87 (61.6)		80 (73.4)	78 (71.6)	
B	130 (28.4)	38 (30.4)		29 (26.6)	31 (28.4)	
ALT (> 40 U/L)	83 (18.1)	23 (18.4)	1	12 (11.0)	21 (19.3)	0.131
AST (> 40 U/L)	104 (22.7)	27 (21.6)	0.896	20 (18.3)	25 (22.9)	0.503
AFP (> 20 μg/L)	196 (42.7)	52 (41.6)	0.905	41 (37.6)	46 (42.2)	0.58
Tumor size (≥ 3 cm)	29 (6.3)	12 (9.6)	0.282	10 (9.2)	11 (10.1)	1
Tumor number (multiple)	92 (20.0)	14 (11.2)	0.032	14 (12.8)	14 (12.8)	1
Subcapsular tumor location (yes)	132 (28.8)	54 (43.2)	0.003	43 (39.4)	49 (45.0)	0.493
Perivascular tumor Location (yes)	27 (5.9)	9 (7.2)	0.739	14 (12.8)	8 (7.3)	0.261
Initial treatment			< 0.001			0.588
Thermal ablation	85 (18.5)	57 (45.6)		47 (43.1)	50 (45.9)	
Surgery	329 (71.7)	57 (45.6)		46 (42.2)	48 (44.0)	
TACE	45 (9.8)	11 (8.8)		16 (14.7)	11 (10.1.1)	
Last tumor			< 0.001			0.782
Primary HCC	372 (81)	46 (36.8)		42 (38.5)	45 (41.3)	
rHCC	87 (19.0)	79 (63.2)		67 (61.5)	64 (58.7)	
Time to recurrence from previous treatment (< 1 year)	214 (52.5)	71 (56.8)	0.452	48 (44.0)	56 (51.4)	0.343

Data were numbers of nodules, with percentages in parentheses

*rHCC* recurrence hepatocellular carcinoma, *RFA* radiofrequency ablation, *PSM* propensity score matching, *LTP* local tumor progression, *IDR* intrahepatic distant recurrence, *TACE* transcatheter arterial chemoembolization

Significant differences in OS were observed between groups based on tumor number, last tumor category, subcapsular location, and previous treatment, prompting PSM to address these imbalances. After PSM, 109 matched pairs were analyzed, showing comparable baseline characteristics (Table 1).

### Technique success rate and major complication

Among all rHCC cases, the technical success rate was 99.1%, with no significant difference between the IDR (457/459, 99.6%) and LTP (122/125, 97.6%, *p* = 0.068) groups. Three of the five patients with residual tumors after RFA received repeated RFA, while the other two received rescue PEI. Complete ablation was ultimately achieved in all cases.

One patient with diabetes in the IDR group had a liver abscess seven days following RFA, followed by toxic shock; the patient finally improved in the intensive care unit, and was discharged. The remaining patients did not experience any severe treatment-related complications.

### LTP

**Fig. 2:**
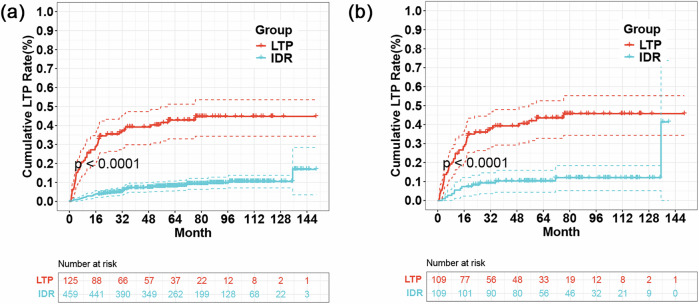
Cumulative curves of LTP in the entire cohort (**a**) and the PSM cohort (**b**). LTP, local tumor progression; IDR, intrahepatic distant recurrence

During follow-up, the estimated cumulative LTP rates at 1 year, 3 years, 5 years, and 10 years were significantly higher in the LTP group than in the IDR group in both the total cohort (2.83%, 7.27%, 8.13%, and 10.51% vs 25.60%, 39.20%, 42.83%, and 44.80%, respectively; *p* < 0.001) and PSM cohorts (5.50%, 10.28%, 10.28%, and 12.04% vs 24.77%, 39.30%, 43.52%, and 45.78%, respectively; *p* < 0.001). (Fig. 2) The LTP ratio in the LTP group was significantly higher than that in the IDR group in both the total (52/125, 41.6% vs 42/459, 9.2%, *p* < 0.001) and PSM cohorts (46/109, 42.2% vs 14/109, 12.8%, *p* < 0.001). (Table 2) Regarding location, 64.2% (36/52) of cases occurred in the original ablation zone (repeated LTP) in the LTP groups, which was significantly higher than in the IDR groups (7.1%, 3/42, *p* < 0.001).

**Table 2:** Treatment response of LTP or IDR after RFA

Group	Before PSM				After PSM			
	No. of patients	LTP	IDR	Extrahepatic metastasis	No. of patients	LTP	IDR	Extrahepatic metastasis
Total	584	94 (16.1)	352 (60.3)	124 (21.2)	218	60 (27.5)	132 (60.6)	53 (24.3)
IDR	459	42 (9.2)	286 (62.3)	92 (20.0)	109	14 (12.8)	67 (61.5)	23 (21.1)
LTP	125	52 (41.6)	66 (52.8)	32 (25.6)	109	46 (42.2)	65 (59.6)	30 (27.5)
*p* value	…	< 0.001	0.054	0.178	…	< 0.001	0.782	0.269
Previous treatment								
Thermal ablation	142	35 (24.6)	82 (57.7)	27 (19.0)	97	30 (30.9)	54 (55.7)	21 (21.6)
Surgery	386	56 (14.5)	237 (61.4)	87 (22.5)	94	28 (29.8)	61 (64.9)	25 (26.6)
TACE	56	3 (5.4)	33 (58.9)	10 (11.9)	27	2 (7.4)	17 (63.0)	7 (25.9)
*p* value	…	0.001	0.732	0.551		0.043	0.412	0.712
Previous tumor								
Primary HCC	418	49 (11.7)	248 (59.3)	88 (21.1)	87	21 (24.1)	46 (52.9)	25 (28.7)
rHCC	166	45 (27.1)	104 (62.7)	36 (21.7)	131	39 (29.8)	86 (65.6)	28 (21.4)
*p* value	…	< 0.001	0.460	0.866		0.362	0.059	0.215

Data were numbers of nodules, with percentages in parentheses

*LTP* local tumor progression, *IDR* intrahepatic distant recurrence, *RFA* radiofrequency ablation, *PSM* propensity score matching, *TACE* transcatheter arterial chemoembolization, *rHCC* recurrence hepatocellular carcinoma

Additionally, rHCC arising from prior rHCC showed a higher LTP risk than from primary HCC (45/166, 27.1% vs 49/418, 11.7%, *p* < 0.001). The LTP rates also differed significantly based on prior treatment. Before PSM, the LTP rates were 24.6% (35/142), 14.5% (56/386), and 5.4% (3/56) in thermal ablation, surgery, and TACE subgroups, respectively (*p* < 0.001). After PSM, the rates were 30.9% (30/97), 29.8% (28/94), and 7.4% (2/27), respectively (*p* = 0.043). The post-RFA LTP rate in the thermal ablation group was significantly higher than in the TACE and surgery groups (Table 2).

### New lesion and extrahepatic metastasis

New intrahepatic tumors were detected in 62.3% and 52.8% of patients in the IDR and LTP groups, respectively (*p* = 0.054), with extrahepatic metastases occurring in 20.0% and 25.6% of these groups, respectively (*p* = 0.178) (Table 2). In the LTP group, the most common metastatic sites, in order, were lung (28.1%), lymph node (15.6%), chest wall (12.5%), and others (bone, diaphragm, paranephros, peritoneum and spleen); while for the IDR, the most common metastatic site was lung (28.3%), followed by peritoneum (13.0%), bone (10.9%), and others (paranephros, lymph node, naps, diaphragm, kidney, pancreas, spleen, brain, stomach and spermatic cord). Previous treatment methods and lesion categories had no significant effect on the occurrence of new lesions or extrahepatic recurrence.

The prevalences of new intrahepatic tumors (67/109, 61.5% vs 65/109, 59.6%, *p* = 0.782) and extrahepatic metastases (23/109, 21.1% vs 30/109, 27.5%, *p* = 0.269) were not significantly different between the IDR and LTP groups after PSM (Table 2).

### Survival outcomes

**Fig. 3:**
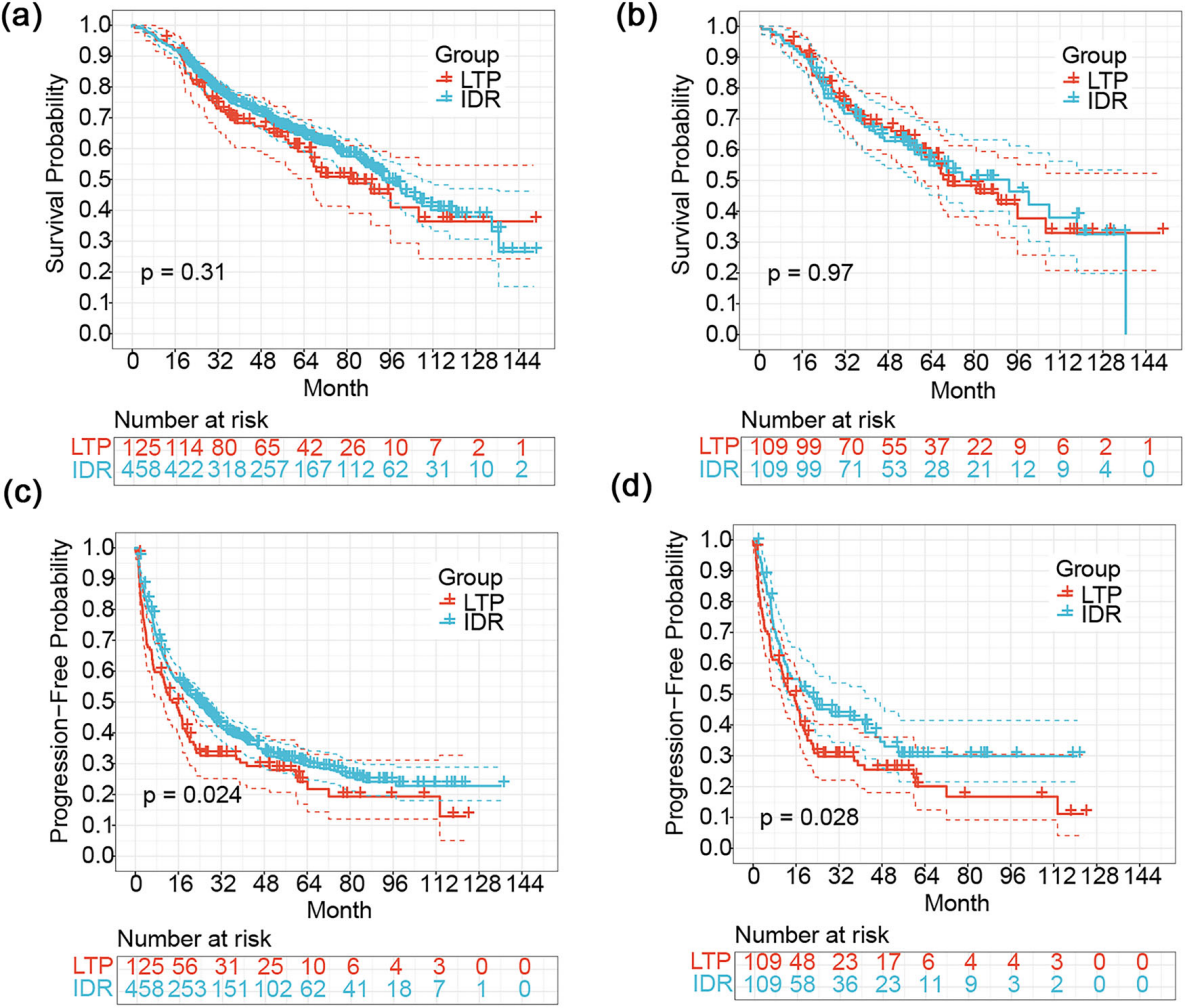
Kaplan–Meier curves for OS and PFS in the entire cohort (**a**, **c**) and the PSM cohort (**b**, **d**), respectively. LTP, local tumor progression; IDR, intrahepatic distant recurrence

The median follow-up time for all patients was 69.5 months. For the total cohort, the median OS time for LTP patients was 80.8 (59.4–102.3) months, compared with 93.3 (83.9–102.7) for IDR patients (*p* = 0.288) (Fig. 3a). The estimated cumulative 1-, 3-, 5-, and 10-year OS rates were 94.12%, 75.17%, 63.71%, 37.52% for the IDR; and 94.39%, 69.32%, 60.47%, 36.42% for the LTP groups, respectively (*p* = 0.305). After PSM, the median OS time for IDR patients was 93.1 (58.0–128.2) months, and 70.3 (51.4–89.2) for LTP patients (*p* = 0.974) (Fig. 3b). The estimated cumulative 1-, 3-, 5-, and 10-year OS rates were 93.58%, 69.58%, 56.82%, 32.54% in the IDR group; and 94.49%, 69.59%, 59.20%, 33.00% in the LTP group, respectively (*p* = 0.974).

The median PFS time for patients in the LTP group was 13.8 (8.6–19.0) months, compared to 23.2 (18.3–28.1) months for patients in the IDR group (*p* = 0.022) before PSM (Fig. 3c). The estimated cumulative PFS rates at 1, 3, 5, and 10 years were 62.51%, 38.72%, 30.40%; and 22.80% in the IDR group, and 52.32%, 32.60%, 24.19%, 12.90% in the LTP group, respectively (*p* = 0.024). After PSM, the median PFS time was 13.8 (9.0–18.5) months of LTP vs 20.9 (9.0–32.8) months of IDR (Fig. 3d), the estimated cumulative 1-, 3-, and 5-year PFS rates were 59.64%, 41.68%, and 29.89% in the IDR group; and 52.65%, 29.78%, and 20.10% in the LTP group, respectively (*p* = 0.028). Due to the limited number of patients with a PFS of > 10 years during the follow-up period, a comparison of the 10-year PFS between the groups was not possible.

**Fig. 4:**
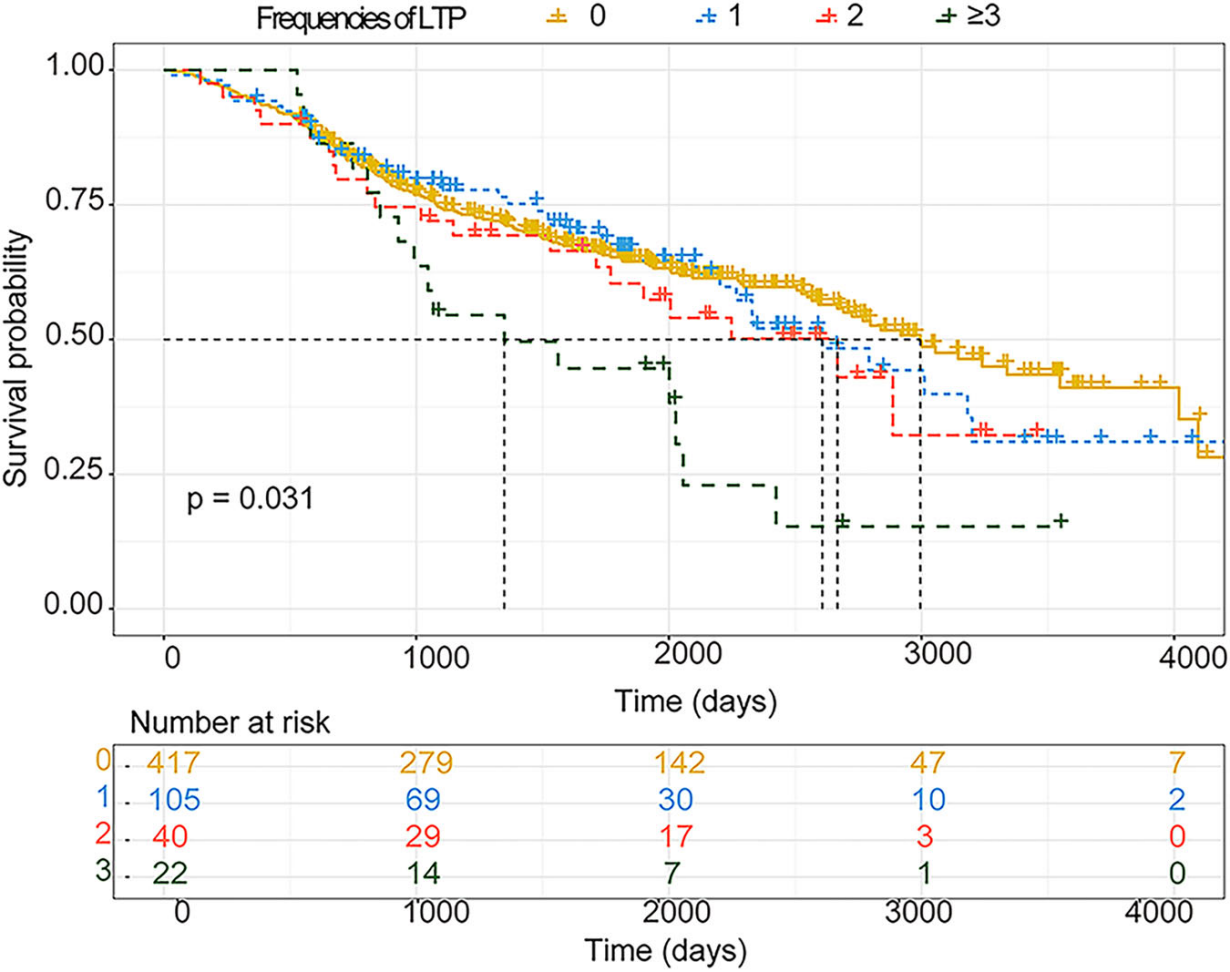
Kaplan–Meier curves in patients with rHCC after RFA with different frequencies of LTP

The median OS times for rHCC patients with frequencies of LTP of 0, 1, 2, and ≥ 3, were 99.3 (87.5–112.2), 86.9 (68.0–105.8), 88.9 (54.4—123.4), and 44.9 (19.9–69.93) months, respectively (*p* = 0.031). Compared with the non-LTPs, those who experienced LTP more than three times present significantly poorer OS (*p* = 0.014) (Fig. 4).

### Factors associated with LTP after RFA in rHCC

Among 584 patients with rHCC, univariate analysis showed that subcapsular location (HR = 1.881, *p* = 0.049), tumor diameter > 3 cm (HR = 2.295, *p* = 0.083), and re-recurrence after rHCC (HR = 6.110, *p* < 0.001) were significant risk factors for repeated LTP after RFA, whereas only recurrence after rHCC (HR = 5.900, *p* < 0.001) was identified in multivariate analysis (Table 3).

**Table 3:** Uni- and multivariable analysis of variables associated with repeat LTP post-RFA in patients with rHCC

Variable	Univariable analysis		Multivariable analysis	
	Hazard ratio	*p* value	Hazard ratio	*p* value
Sex (male)	1.279 (0.536, 3.053)	0.579		
Age (≥ 60 years)	1.238 (0.660, 2.324)	0.506		
HBV (positive)	0.894 (0.215, 3.708)	0.877		
HCV (positive)	1.427 (0.196, 10.395)	0.726		
Anti-hepatitis therapy (yes)	1.469 (0.776, 2.782)	0.237		
Cirrhosis (yes)	0.628 (0.335, 1.183)	0.150		
Portal hepertension (yes)	0.586 (0.269, 1.275)	0.178		
Child–Pugh grade (B)	1.444 (0.751, 2.778)	0.271		
Elevated AFP	1.099 (0.584, 2.070)	0.770		
Previous treatment (thermal ablation vs surgery vs TACE)	1.002 (0.837, 1.198)	0.987		
Previous tumor (rHCC vs HCC)	6.110 (3.095, 12.063)	< 0.001	5.900 (3.000, 12.000)	< 0.001
Tumor size (> 3 cm)	2.295 (0.897, 5.873)	0.083	2.500 (0.960, 6.500)	0.060
Tumor number (multiple vs single)	1.020 (0.450, 2.311)	0.962		
Subcapsular tumor location (yes)	1.881 (1.002, 3.531)	0.049	1.400 (0.740, 2.700)	0.290
Perivascular tumor Location (yes)	1.749 (0.622, 4.922)	0.289		
Early recurrence from previous treatment (≤ 1 year)	1.351 (0.714, 2.558)	0.356		

Data in parentheses are 95% CIs

*LTP* local tumor progression, *IDR* intrahepatic distant recurrence, *RFA* radiofrequency ablation, *rHCC* recurrence hepatocellular carcinoma, *AFP* *α*-fetoprotein, *TACE* transcatheter arterial chemoembolization

### Factors associated with PFS and OS after RFA in rHCC

The results of the univariate and multivariate analyses of PFS and OS were presented in Table 4. Cirrhosis (HR = 1.500, *p* = 0.003), portal hypertension (HR = 1.200, *p* = 0.024), AFP > 20 ng/mL (HR = 1.500, *p* = 0.002), re-recurrence after rHCC (HR = 1.300, *p* = 0.035), tumor diameter > 3 cm (HR = 3.200, *p* < 0.001), multiple tumors (HR = 2.200, *p* < 0.001), subcapsular tumor (HR = 1.100, *p* = 0.056), and recurrence within 1 year (HR = 2.200, *p* < 0.001) were independent risk factor for OS. AFP > 20 ng/mL (HR = 1.300, *p* = 0.014), recurrence after rHCC (HR = 1.700, *p* = 0.062), multiple tumors (HR = 1.700, *p* < 0.001), and recurrence within 1 year (HR = 1.500, *p* < 0.001) were independent risk factors for PFS.

**Table 4:** Uni- and multivariable analysis of variables associated with PFS and OS post-RFA in patients with rHCC

Variable	PFS				OS			
	Univariable analysis		Multivariable analysis		Univariable analysis		Multivariable analysis	
	Hazard ratio	*p* value	Hazard ratio	*p* value	Hazard ratio	*p* value	Hazard ratio	*p* value
Sex (male)	1.134 (0.831, 1.546)	0.428			1.248 (0.836, 1.864)	0.278		
Age (≥ 60 years)	0.982 (0.803, 1.201)	0.860			1.083 (0.839, 1.399)	0.540		
HBV (positive)	0.998 (0.622, 1.602)	0.994			0.789 (0.460, 1.354)	0.390		
HCV (positive)	0.589 (0.244, 1.422)	0.239			1.304 (0.537, 3.164)	0.558		
Anti-hepatitis therapy (yes)	1.049 (0.861, 1.277)	0.636			1.107 (0.860, 1.425)	0.430		
Cirrhosis (yes)	0.990 (0.812, 1.207)	0.923			1.359 (1.050, 1.758)	0.020	1.500 (1.000, 2.000)	0.003
Portal hypertension (yes)	1.081 (0.873, 1.339)	0.473			1.336 (1.024, 1.742)	0.033	1.200 (0.890, 1.600)	0.024
Child–Pugh grade (B)	1.059 (0.851, 1.317)	0.610			1.122 (0.847, 1.486)	0.424		
Elevated ALT	1.071 (0.832, 1.379)	0.595			1.258 (0.928, 1.707)	0.139		
Elevated AST	1.063 (0.841, 1.344)	0.608			1.195 (0.891, 1.603)	0.234		
Elevated AFP	1.345 (1.103, 1.640)	0.003	1.300 (1.100, 1.600)	0.014	1.513 (1.175, 1.949)	0.001	1.500 (1.200, 1.900)	0.002
Previous treatment (thermal ablation vs surgery vs TACE)	1.002 (0.837, 1.198)	0.987			1.133 (0.897, 1.431)	0.294		
Previous tumor (rHCC vs HCC)	1.314 (1.062, 1.626)	0.012	1.700 (1.300, 2.200)	0.062	1.403 (1.073, 1.836)	0.013	1.300 (1.000, 1.800)	0.035
Tumor size (> 3 cm)	1.266 (0.856, 1.872)	0.237			2.537 (1.727, 3.725)	< 0.001	3.200 (2.200, 4.800)	< 0.001
Tumor number (multiple vs single)	1.719 (1.349, 2.191)	< 0.001	1.700 (1.300, 2.200)	< 0.001	1.953 (1.458, 2.617)	< 0.001	2.200 (1.700, 3.000)	< 0.001
Subcapsular tumor location (yes)	1.047 (0.848, 1.293)	0.667			1.268 (0.972, 1.653)	0.080	1.100 (0.830, 1.400)	0.056
Perivascular tumor location (yes)	1.034 (0.684, 1.563)	0.873			1.391 (0.880, 2.199)	0.158		
Early recurrence from previous treatment (≤ 1 year)	1.508 (1.236, 1.839)	< 0.001	1.500 (1.200, 1.800)	< 0.001	2.161 (1.651, 2.829)	< 0.001	2.200 (1.700, 2.900)	< 0.001

Data in parentheses are 95% CIs

*LTP* local tumor progression, *IDR* intrahepatic distant recurrence, *RFA* radiofrequency ablation, *rHCC* recurrence hepatocellular carcinoma, *ALT* alanine aminotransferase, *AST* aspartate aminotransferase, *AFP* *α*-fetoprotein, *TACE* transcatheter arterial chemoembolization

## Discussion

This study reported a retrospective analysis to compare long-term outcomes after RFA in rHCC patients with LTP vs those with IDR. Overall, RFA demonstrated comparable long-term OS between the LTP and IDR groups (*p* = 0.974). Nevertheless, PFS was inferior in the LTP group (*p* = 0.028), predominantly due to a markedly higher cumulative LTP rate (*p* < 0.001). Additionally, patients who experienced three or more LTP events had significantly poorer OS compared with those who had 0–2 LTP episodes (*p* = 0.031).

Although RFA is widely used for rHCC, our findings highlight that its long-term efficacy differs between recurrence patterns. A recent meta-analysis [27] of 16 studies reported broad ranges in 3- and 5-year RFS rates (12.4–45.1% and 0–78.9%), and OS rates (48.6–85.7% and 20–84.9%), within which our cohort’s outcomes fall. After PSM, key baseline characteristics, including tumor size, location, recurrence history, and underlying liver function, were comparable between groups. Despite comparable OS, PFS was significantly inferior in the LTP group compared to the IDR group, primarily due to the higher incidence of subsequent LTP rather than new intrahepatic lesions or extrahepatic metastasis. Specifically, LTP occurred in 41.6% of patients in the LTP group compared with only 8.7% in the IDR group. These findings underscore that the higher recurrence risk in LTP necessitates more precise ablation strategies and intensified post-RFA surveillance.

Previous studies have shown that RFA could achieve a prognosis comparable to surgery or TACE [14–17]. The reported 1-, 3-, and 5-year RFS rates were 41.7–57.2%, 13.2–28.6%, and 8.8–17%, respectively. In our cohort, the 1-, 3-, 5-, and 10-year RFS rates were 52.6%, 29.8%, 20.1%, and 11.2%, respectively—broadly consistent with existing literature. However, these studies did not further investigate the clinical management or prognostic implications of repeated LTP episodes, which remains a critical gap in clinical practice. In the present study, one or two LTP episodes did not significantly impact OS, whereas three or more LTP episodes were associated with significantly poorer survival. Regarding risk factors for LTP following RFA in rHCC, while previous studies have emphasized tumor size and subcapsular location [28], our analysis identified multiple tumor recurrences as a more significant predictor. Supporting this, prior research has shown that during HCC recurrence, subclone-driven mutations and biological alterations, including epithelial-mesenchymal transition, autophagy activation, non-coding RNA dysregulation, and tumor microenvironment changes, may enhance heat resistance, despite effective control of imaging-visible rHCC [29–31]. This may be one of the important reasons for poorer prognosis with increasing LTP episodes. Additionally, patients undergoing multiple ablations may also experience psychological distress due to frequent relapses, hospitalization-related stress, and reduced cost-effectiveness. Therefore, for patients experiencing ≥ 3 LTP episodes, alternative radical strategies such as salvage surgery, stereotactic body radiotherapy, or systemic therapy should be actively considered even if ablation remains technically feasible.

This study also identified liver cirrhosis, portal hypertension, AFP > 20 ng/mL, tumor diameter > 3 cm, multiple tumors, and subcapsular location as independent predictors for poor OS, which aligns with prior reports [7, 32, 33]. Although early recurrence after hepatectomy is a well-established indicator of poor OS [34], the impact of early recurrence after RFA has rarely been examined, and our findings contribute to this area.

These findings highlight that, beyond traditional clinical staging, the patient’s specific history of recurrence pattern should be considered, and treatment stage migration [3] should be considered in cases of multiple recurrences, early recurrences, or repeated LTP to optimize outcomes.

Our study has several limitations. First, the retrospective single-center design may have introduced a selection bias. Second, the patients received various treatments for HCC before RFA, which is a significant variable; however, we applied PSM to minimize bias. Third, despite the high complete ablation rate after RFA, the long study period may have led to variations in techniques and assessments, potentially causing bias.

In conclusion, this study shows that RFA is effective in managing both LTP and IDR, with a similar OS. However, PFS was poorer after LTP ablation because of the higher LTP risk. Key OS risk factors include liver cirrhosis, portal hypertension, AFP levels > 20 ng/mL, tumor diameter > 3 cm, multiple and subcapsular tumors, frequent recurrence, and early recurrence. Notably, ≥ 3 LTP episodes were identified as a substantial risk factor for OS.

## Data Availability

All data supporting the findings are available from the corresponding author on reasonable request.

## Abbreviations

IDR: Intrahepatic distant recurrence
LTP: Local tumor progression
OS: Overall survival
PEI: Percutaneous ethanol injection
PFS: Progression-free survival
PSM: Propensity score matching
RFA: Radiofrequency ablation
rHCC: Recurrent hepatocellular carcinoma
TACE: Transcatheter arterial chemoembolization
US: Ultrasound
